# Health of Towns' Association. Report of the Committee to the Members of the Association on Lord Lincoln's Sewerage, Drainage, &c. Of Towns Bill

**Published:** 1847-01-01

**Authors:** 


I. Health of Towns' Association. Report of the Committee to the Members of the Association, on Lord Lincoln's Sewerage, Drainage, &c. of Towns' Bill. Pp. 122. London: Charles Knight and Co. 1846.

II. Unhealthiness of Towns, its Causes and Remedies; being a Lecture delivered in the Mechanics' Institute at Plymouth. By Viscount *Ebrington*, M.P. Published by the Health of Towns' Association. London: Charles Knight and Co. 1846.

III. On the Health of Towns, as influenced by Defective Cleansing and Draining, and on the Application of the Refuse of Towns to Agricultural Purposes. Being a Lecture delivered at the Russell Institution. By *William A. Guy*, M.B. Cantab., Professor of Forensic Medicine, King's College, &c. London: Henry Renshaw, 1846.

IV. The Liverpool Health of Towns' Advocate; published under the Sanction of the Committee of the Liverpool Health of Towns' Association. Edited by *John Sutherland*, M.D. Senior Physician to the Liverpool Dispensaries. London: Longman and Co. 1846.

V. The Improvement of Manchester. A Report setting forth a Plan proposed by the Towns' Improvement Company, communicated to the General Purposes' Committee of the Town Council.

Of these several publications, the most important is the Report lately issued by the Committee of the Metropolitan Health of Towns' Association; and when we announce that this valuable document emanates from the pen of Dr. Southwood Smith, it would be superfluous to add that it is deserving of the most careful consideration of all who are interested in the great question to which it relates.

In a former Number of this Journal (see Med.-Chir. Rev., Oct. 1845) a hope was expressed that, ere this, legislative means would have been devised to rectify the crying evils, which afflict the poorer part of the inhabitants of our large towns and populous districts. Unhappily, however, this all-important question has, like so many others affecting social improvement, been deferred amidst that endless conflict of parties, classes, and sects, which well nigh defeats one of the great objects of all government—the promotion, namely, of the physical, moral, and religious welfare of the people. In one respect the delay has been advantageous, inasmuch as it has afforded an opportunity of maturely considering the merits of the measure introduced by the late Government, and commonly known as Lord Lincoln's Bill. This important and somewhat difficult task has been very fitly undertaken by the Health of Towns' Association, a society which comprises among its committee some of the most distinguished and consistent advocates of sanitary improvement, including, with many members of the two houses of parliament, several eminent individuals connected with our own profession. It is also satisfactory to perceive that those two great cities, Manchester and Liverpool, which are so fearfully interested in this question, are actively putting forth their energies to struggle with the gigantic evils by which, in common with all other populous places, they are so sorely afflicted. Nor can it be otherwise than highly gratifying, that one of the nobles of the land, Lord Ebrington, assuming the functions of an instructor of the people, has efficiently advocated this great cause by addressing a popular audience in a large provincial town. These must all be regarded as favourable indications; because they sufficiently show that, however lukewarm or indifferent our rulers may hitherto have been, the indispensable necessity of a general and comprehensive change in the whole system of drainage, supply of water, ventilation, and other similar matters, has become universally recognised.

But beyond all other circumstances of promise is the unquestionable fact that we have at last a *willing Government;* so that whatever other difficulties may remain to be surmounted, and these are neither insignificant in number nor unimportant in character, the main obstacle to a thorough amendment has at all events been removed—a ministry, namely, entering upon one of the most vital questions ever brought before the legislature of this country, actuated rather by a concern to preserve vested rights than by an anxious desire to ameliorate the condition of the industrial classes. Favourable as are thus the prospects of a speedy and effectual amendment, and zealous as are the exertions now being made in so many parts of England; it is yet a point of momentous consequence, that no erroneous or short-sighted principles should be allowed to prevail, nor that any false step should be taken in a matter so nearly concerning the lives and happiness of millions. It is with this feeling and with the knowledge that medical men must and ought to have a powerful voice in this question, that we propose to lay before our readers such an abstract of the above-named "Report," as will convey to them the matured views of those best qualified to judge of what is required to constitute a comprehensive plan of sanitary improvement.

In our former article some of the defects of the Government measure were pointed out; and we especially showed the absurdity and injustice of excluding the metropolis from the benefits of the bill; an omission, we imagine, dictated by something of the same kind of feeling, which, in the matter of corporation reform, stayed the hands of a Whig administration when they approached the colossal abuses of the city. In the Report before us we find the following paragraph relative to this and other equally important omissions.

"The first point to which your Committee would direct attention is the proposed limitation of the Act: that is, the limitation of its operation to England and Wales to the exclusion of Ireland and Scotland, and to the further exclusion of the metropolis even of England itself. Now a large proportion of the evidence on which the conclusions of the first and second Reports of Her Majesty's Commissioners are founded is derived from the experience of the metropolis, and the specific recommendations of the second Report are applied directly to evils prevalent in the metropolis; while it is universally known and admitted that the towns in Ireland and Scotland are subject to the same evils as those ascertained to exist in the English towns, only commonly in greater intensity, and that consequently they stand in still more pressing need of remedies." P. 6.

In an interview which lately took place between the Home Secretary and a deputation from the Health of Towns' Association, an assurance was given that one of these glaring defects should be rectified by including the metropolis in the new sanitary measures about to be introduced by the present Government. This is so far well; but the duty of the legislature will be only half performed, if two of the component parts of the Empire, Ireland and Scotland, with their millions of inhabitants, are deprived of the inestimable benefits which must accrue from any well-devised scheme of improvement. We are also ourselves convinced that the rural districts of England urgently require ameliorations, which, if left to the apathetic ignorance and short-sighted selfishness of the parties who exercise the principal influence in those localities, will be indefinitely postponed. There is at this time a vast amount of preventible disease occurring in villages and small towns, causing from time to time frightful ravages; and often entailing upon the agricultural labourers and their families, years of subsequent distress, owing to the permanent debility induced by typhoid fevers. And yet, in these very districts, the happiest results have invariably followed even imperfect sanitary measures, such as filling up stagnant ponds in the centre of villages, covering over open ditches, and similar proceedings. There is also an element of success in these instances of primary consequence—the facility, namely, of carrying out the requisite improvements. In large towns, the vast amount of the existing evils will, under the best-directed efforts, offer a very serious impediment; but, in the country, a comparatively trifling expenditure would realise all that was required, and would speedily be repaid by the saving effected in the poor-rates. We have been given to understand that the Health of Towns' Association propose to institute, through the instrumentality of one of its members, an inquiry into this important subject, with the view of ascertaining what is the existing amount of preventible sickness in rural districts, and what have been the results of sanitary improvements where these have been introduced.

After pointing out the ignorance and extravagant jobbing of some of the existing boards, as well as the obstructions to sanitary improvement caused by these bodies, the Westminster Court of Sewers being selected for illustration, the Report points out a serious error in Lord Lincoln's bill, which, "instead of charging the responsible (public) officer with the duty of preparing in all cases plans and estimates, expressly empowers the Commissioners (a fluctuating body proposed to be constituted by the bill, without professional knowledge and irresponsible) to commence and execute works." As if to perpetuate the very abuses for the abolition of which all disinterested parties are seeking, the bill of the late Government does not adopt the recommendation of the Commissioners of Inquiry, that all works whatever should be executed by contracts upon open tenders. It is needless to point out further that, without such a provision, the public can never be guarded from jobbing, extravagance, and incompetency.

It is a remarkable illustration of the slow march of improvement in legislation, that whereas there is an universal conviction from one end of the kingdom to the other, that if the supply of that prime essential of health, water, is to be efficient it must be constant, and upon the principle of "high pressure," the bill in question, recognises and perpetuates the old intermittent supply, with all its costly and clumsy encumbrances of cisterns, water-butts, ball and stop-cocks; a system condemned without exception by all who have investigated the subject, as being injurious to the health and morals of the labouring classes; as vitiating the water, curtailing the supply, and putting the consumer to unnecessary trouble, inconvenience and expense. We would call the earnest attention of those who are occupied in so many towns of England in devising plans for their improvement, to the following extract from the Report, which concludes the admirable review of the whole subject; and which is particularly applicable at the present moment, since we are ourselves acquainted with more than one town, where the interested opposition of a few powerful individuals, interested in water companies, threatens, if not to arrest the progress of improvement, at least to inflict on the rate-payers a heavy pecuniary sacrifice in legal expenses.

"Your Committee submit that the facts and reasonings now adduced completely establish the case for which they thus earnestly contend, namely, that every water company seeking legislative aid and protection ought to be compelled by the legislature to furnish the public with filtered water at constant service and high pressure. But if it is right that the legislature should require all existing water companies to submit to this condition, and to whatever other regulations are proved to be necessary for the maintenance of the public health and the protection of public property, *à fortiori* it ought to withhold its sanction from the establishment of new water companies until the provisions are determined on which the powers and privileges sought can be granted without compromising the interests of the community. Her Majesty's Commissioners state, as the conclusions to which they have arrived from the vast mass of evidence that has come before them, that the water supply should be constant; that it should be consolidated with the works of drainage; that to separate these works is to render both inefficient, and to double or treble the expense for the construction, maintenance, and working of each. The Commissioners will therefore, indeed, have laboured in vain, if the legislature should listen to the applications now before Parliament; applications which the very announcement of the conclusions of the Commissioners appear to have suggested; schemes for the pre-occupation of towns by water-works for trading companies, without the slightest additional security for the public interests.

"A regulation appears, indeed, to have been adopted during the last session of Parliament, that the water companies then sanctioned should be subjected to any future regulations that might hereafter be made in respect to such companies. But the subjection of these companies to future regulations will not prevent them from making large claims for compensation for the alteration of their first arrangements."—*Report*, p. 64–5.

The abolition of those unseemly and demoralising abominations, cesspools and privies, and the substitution of self-acting water-closets, have been proved by a vast amount of evidence to be economical, as well as highly conducive to health, decency, and morality; and yet in Lord Lincoln's bill the present debasing system is perpetuated. There can, we conceive, be no dissent from the opinion expressed in this paragraph: "These and similar statements have satisfied your Committee that legislation on this point ought to be in accordance with the evidence, and with the recommendations of Her Majesty's Commissioners; that the construction of the cesspool in all new dwellings ought to be positively prohibited; and that the removal of all existing cesspools ought to be made compulsory, as soon as the general introduction of sewers and drains, combined with an ample supply of water, shall have rendered the general introduction of the water-closet apparatus practicable."—*L. c.*, p. 71.

Another most remarkable defect is that of ventilation, which is altogether omitted, the bill "resembling in this respect those modern encyclopædias of architecture and building, in which the very word ventilation never once occurs from beginning to end." Although it is impossible to overrate the advantages which would result from the general introduction of efficient ventilation in the houses of the poor, yet it would be inexpedient to attempt this by compulsory provisions, as these would interfere with the privacy of domestic life. "But this objection does not apply to buildings intended for public resort, such as churches, courts of justice, concert and assembly-rooms, theatres, houses and rooms for the public use of which a licence is required; factories already under government regulation and inspection; workshops in which great numbers of workpeople habitually assemble, lodging-houses, and schools. The introduction in a general sanatory measure, of compulsory provisions for the purpose of securing proper ventilation in all places of this description, appears to be justified by the absolute necessity of the case, and to be free from any objection in principle."—*L. c.*, p. 79.

In alluding to the classes defining the duties of the officer of health, as set forth in the bill formerly laid before Parliament, the Report before us contains the following judicious remarks.

"The duties assigned to the medical officer of health in the first of these clauses are highly important, and the able performance of them throughout the country will produce beneficial results, the true value of which it is impossible at present to estimate. Still, however, these provisions do not go to the root of the matter, nor embrace the primary and fundamental duties of the officer of health. These fundamental duties are the verification of the fact as well as of the cause of death, the correct registration of both, and the personal examination on the spot of the sanatory circumstances under which death takes place. It is only by the performance of these primary duties that the duties described in the Bill can be properly performed; that the existence and prevalence of diseases can become known; that the local causes originating and maintaining such diseases can be traced; that the most efficacious modes of checking or preventing their spread can be ascertained; and, consequently, that a true report on the sanatory condition of any town or district can be framed."—*L. c.*, p. 91.

If such officers had existed, the frightful scenes described as often occurring in the abodes of the poor in large towns, when one of their number is struck by death, could never have arisen. One or two instances will suffice to show how urgently this subject calls for the interposition of the legislature. "There are some houses in my district," says Mr. Leonard, surgeon, one of the medical officers of the parish of St. Martin's in the Fields, "that have from forty-five to sixty persons of all ages under one roof; in the event of death, the body often occupies the only bed, till they raise money to pay for a coffin, which is often several days. * * * * * * Upon the 18th of December, 1840, I—— and her infant were brought ill with fever, to her father's room in Eagle-court, which was ten feet square, with a small window of four panes ; the infant soon died. Upon the 15th January, 1841, the grandmother was taken ill: upon the 2nd of February the grandfather also. There was but one bedstead in the room. The corpse of the grandmother lay beside her husband upon the same bed, and it was only when he became delirious and incapable of resistance (on account of his violent objection to his own removal and that of the dead body from the room) that I ordered the removal of the body to the dead-house, and him to the Fever Hospital. He died there, but the evil did not stop here: two children, who followed their father's body to the grave, were, the one within a week and the other within ten days, also victims to the same disease. In short, five out of six died." The following case is taken from the evidence of Mr. Wild, an undertaker.

"The other day at Lambeth the eldest child of a person died of scarlet fever; the child was four years old: it had been ill a week; then came two other children, one three years and the other sixteen months old. When the first child died there were no symptoms of illness for three days afterwards; the corpse of the eldest one was in a separate room; but the youngest child had been taken by the servant into this room ; this child was taken ill and died in a week. The corpse was retained in the house three weeks, at the end of which time the other child also died."—*L. c.*, p. 95.

We believe it is impossible to exaggerate the beneficial results which may be anticipated from the investigation of disease with a view to prevention. The author of the Report we are noticing, justly observes, " man has but little power over the progress of disease when once it is produced ; but he may exercise a very important control over the circumstances and conditions that give origin to it, when those circumstances and conditions are once ascertained." (*L. c.*, p. 101.) To know with certainty the cause of a disease, is very often to prevent its occurrence. Do we not see this in the case of intermittent fevers ; do we not know that effectually to drain a marshy district, is to eradicate the ague formerly prevailing ? And can it be doubted that were the diseases characteristic of our crowded towns thoroughly investigated, they would, in like manner, by removing the cause, be also exterminated ? One, then, of the chief objects to which the attention of the officer of health should be directed is " *the science of prevention.*" After noticing the advantages derived from the systematic observations made by military and naval surgeons on large bodies of men, the writer of this Report goes on to affirm ; " but in towns and cities large classes of persons often exist under conditions as well defined and as steady in their operation as the circumstances presented to the observation of medical officers of the army and navy ; the knife-grinders of Sheffield, for example, the ironmongery and toy manufacturers of Wolverhampton, the persons employed in particular departments of colliery and factory labour, persons who work together in large numbers in common workshops, as tailors, dressmakers, &c. In each of these cases, and there are many others, the circumstances injurious to health are common to great numbers : they are steady in their operation, they are uniform in their result ; the connexion between cause and effect can be clearly traced, and in this manner the efficacy of some particular remote cause in producing some peculiar form of disease may be determined statistically and with absolute certainty, and knowledge of the highest importance may be thus acquired, leading directly and certainly to the prevention of disease. What additions may be made to our knowledge of these causes and of the means of counteracting and removing them by the combined and continued labours of such a body of public servants, it is impossible to predict; but surely these observations indicate a new direction in which protection of the highest kind may be extended to the community, and especially to the poorer classes, that well deserves the attention of the statesman."—*L. c.*, p. 102.

We have now noticed in some detail the leading branches of this great question; but in order to present in one view what should be the principles guiding an enlightened legislative enactment, we are anxious to lay before our readers the general conclusions with which Dr. Smith concludes his valuable Report; and which, although taking the form of critiques upon Lord Lincoln's bill, embody in reality the whole bearings of the sanitary question.

The sound provisions of the measure are affirmed to be as follows:—

"1. The general enactment, that the supply of water, the sewerage, the drainage, the cleansing, and the paving of towns, including the suburbs, shall all be placed under one and the same authority:
2. The appointment of a Government Inspector:
3. The appointment of an Inspector of Nuisances:
4. The appointment of Local Boards of Commissioners for carrying out the provisions of the Act in their respective districts:
5. The preparation or the local examination of surveys, plans, and estimates, by competent and responsible officers, before any works are undertaken:
6. The publication of these surveys, plans, and estimates, with expository Reports for local distribution, in order that the proposed works may be thoroughly canvassed by all parties interested in them before they are commenced:
7. The execution and maintenance of all works by contract; the peformance of the contracts to be supervised by paid and responsible local officers:
8. The appointment in districts of medical officers of health." Pp. 118–19.

The errors and defects of the Bill are so grave that they require to be prominently pointed out.

"1. The limitation of the Act to England and Wales, to the exclusion of the Metropolis even of England, and to the total exclusion of Ireland and Scotland, without providing for the immediate preparation of a survey and plan of the metropolis, and a Report as to the special measures applicable to the Metropolis, to Ireland, and to Scotland:
2. The omission to create a central superintending authority in subordination to the executive government, invested with the same sort of powers with reference to the local Boards intrusted with the execution of the details of the Act, that the Poor Law Commissioners have with the Boards of Guardians; instead of this, giving the entire superintendence of the Act to the Secretary of State for the Home Department:
3. The omission to take adequate powers for compelling the Boards of Local Commissioners duly to execute the Act:
4. The creation of a new, complex, and needless machinery for electing Boards of Commissioners, instead of adopting the mode of electing Boards of Guardians now in use throughout the Poor Law Unions, which is found in practice to work perfectly well:
5. Investing the Boards of Commissioners with powers to execute works, instead of rendering their functions entirely and strictly ministerial and supervising, and neglecting positively to restrict by an express enactment their duties to acts of this class:

6. The omission to prohibit by a sufficiently stringent enactment, Boards of Commissioners from commencing any works without having caused plans and estimates to be prepared by their own surveyor, and without having obtained for these plans and estimates the sanction of the Inspector:
7. The omission to secure by sufficiently stringent enactments that all works whatsoever shall be executed only by contract upon open tenders, and shall be maintained in repair for terms of years; and that the contractor shall be bound to undertake any extraordinary works at a fixed remuneration:
8. The omission to provide facilities for the formation of public companies for carrying out by contract the provisions of the Act:
9. The omission to make sufficient provision for raising the necessary capital for the execution of large sanatory improvements: namely, by loan raised on the security of a special rate to be levied on the properties in the several localities, the principal and interest to be repaid by annual instalments within a limited number of years.
10. Fixing the cost on owners, whereas it ought to be placed on occupiers:
11. Neglecting to provide in the manner above recommended, that the expense remain a charge upon the several properties, unless the owners prefer to pay the cost in the first instance:
12. Neglecting to make it compulsory on water companies to give the public a constant instead of an intermittent supply, and to deliver it in all cases at as high a pressure as is practicable:
13. Neglecting to make it compulsory on water companies either to filter the water or to provide a sufficient area of depositing bed:
14. The omission absolutely to forbid the construction of cesspools in all new dwellings, and to provide for the compulsory removal of all existing cesspools as soon as the general introduction of sewers and drains, combined with an adequate supply of water, shall have rendered the universal adoption of the water-closet apparatus practicable:
15. Neglecting the entire subject of ventilation, one of fundamental importance in a sanatory measure:
16. The omission to give adequate powers to the Commissioners to remove, under the direction of the Inspector and the District Officer of Health, any house or houses which may be so situated as to render a street a cul-de-sac, preventing the possibility of a current of air from passing through it; and, the further omission to give power to the same authorities to raise money for opening thoroughfares, and for the construction and maintenance of public walks:
17. The omission to provide for the removal of nuisances arising from manufactories in towns and populous districts:
18. The omission to provide for the removal of the smoke nuisance:
19. Neglecting in reference to the medical officer of health to make provision for the performance of his primary and essential duties; namely, the verification of the fact as well as of the cause of death, the correct registration of both, and the personal examination on the spot of the sanatory circumstances under which death takes place:
20. The omission to make any modification in the mode of assessment of the window-duties, though a principle of assessment has been pointed out by the adoption of which the revenue need lose nothing, while great facilities would be afforded for the better construction of dwelling-houses, and for the freer admission to them of light and air.

"If the provisions enumerated are passed into a law, and if the errors and omissions pointed out are corrected and supplied, this Act will, in the opinion of your Committee, form one of the most comprehensive, efficient, and beneficent statutes ever enacted by any legislature in any age or country. Its direct effect will be the renovation of the physical strength and vigour of the people, and an augmentation of their means of subsistence, first, by increasing and sustaining their working power, and secondly by diminishing the sum at present expended on sickness, orphanage, and premature decrepitude; and ultimately, a large addition to their longevity: while indirectly but not less certainly it will promote their intellectual, moral, and social improvement. Your Committee, therefore, earnestly request the attention of the members of the Association and of the public generally to the facts and conclusions now stated, and they respectfully submit them to the consideration of the Government and of the Legislature."—*L. c.*, pp. 118–122.

The Liverpool Health of Towns' Association, duly impressed with the painful revelations as respects that great centre of commerce, made in the report of the Commissioners of Inquiry, have zealously and successfully entered upon the good work of improvement; and have set an example which might with great advantage be imitated elsewhere. They have instituted lectures in various parts of the town of Liverpool; they have held public meetings; they have called the attention of the municipal authorities to nuisances and other evils endangering the public health; and, by the publication of a cheap monthly paper, they have laboured to remove error, and to diffuse information, and have thus kept the whole subject alive in the public mind. Efforts such as these are of the first consequence, inasmuch as they not only enlighten the community upon matters in which all have so deep a concern; but likewise, because, by carrying conviction to the minds of the educated classes, they tend to strengthen the hands of the Government; which even when, as at present, favourably inclined, will generally in affairs of this kind, involving such varied and powerful interests, but presenting no political rallying-point, regulate its action to a much greater extent than is ordinarily conceived, by the amount of support it receives from without.

The "Health of Towns' Advocate" contains much valuable matter; and the principles therein expounded have, as the Committee state in their Preface, more than a merely local interest. In Liverpool the causes of insalubrity—over-crowding, filth, bad drainage, damp dwellings, and defective ventilation—act with dire intensity; and the result is, that the three classes of disease, which are the more peculiar index of the amount of preventible sickness—fever, consumption, and convulsions in infants, prevail to a frightful extent. The following table is a melancholy but instructive evidence of this assertion:—

"PROPORTION OF DEATHS, FROM THREE DISEASES, TO THE WHOLE POPULATION ANNUALLY.

| *Diseases.* | *Birmingham.* Deaths. | *Leeds.* Deaths. | *Metropolis.* Deaths. | *Manchester.* Deaths. | *Liverpool Parish.* Deaths. |
|---|---|---|---|---|---|
| Fever . . . . | 1 in 917 | 1 in 849 | 1 in 690 | 1 in 498 | 1 in 407 |
| Consumption . | 1 in 207 | 1 in 209 | 1 in 246 | 1 in 172 | 1 in 166 |
| Convulsions . | 1 in 645 | 1 in 301 | 1 in 453 | 1 in 205 | 1 in 188 |

"We thus find that, in proportion to the population, the deaths from fever are more than double in Liverpool what they are in Birmingham; that above half as many more die from consumption in Liverpool as in London; and that more than three times as many children perish annually from convulsions in Liverpool as in Birmingham."—*Health of Towns' Advocate*, p. 14.

After adducing other proofs of the vast amount of sickness prevalent in this afflicted town, the conductors of the "Advocate" conclude their second number with these most just remarks.

"It is easy to read of these things; but it is not so easy, without personal experience, to realize their full meaning. We are too apt to consider such statements as mere barren statistical results. They have in them, nevertheless, an awful depth of significancy. They are the indexes of a degree of human woe, compared with which many things that move our deepest sympathies are hardly worthy of mention; and of a needless waste of human life, which, whether we consider its continual existence, its extent, or its accompanying sufferings, throws into the shade the slaughter of battle fields. When we think of the dreadful localities in which sickness has to be endured; the absence of even the most needful comforts in illness; the loss of time and wages, which are their only property, on the part of heads of families, and the consequent privation to the families themselves;—the awful mortality, especially amongst the young, from whence it arises that in some instances 64 per cent. of all who die are children under five years of age, while the average age of death of the whole class is reduced to 13½ years;—the heart-breaking sorrow that is itself so powerful an agent in the production of disease;—when these things are considered, and when we remember that the causes of all are to a great extent under our control, it will surely require no argument on our part to form the determination, in every well-constituted mind, never to rest till such evils have come to an end."—*L. c.*, p. 15.

It is consistent with our personal knowledge to state that the authorities of Manchester have for some time been zealously endeavouring to ameliorate the condition of that populous town; and in the Report before us, there is afforded further proof that the Town Council is anxious to adopt the best means of removing the existing evils. After having devoted so much space to this subject, we can only remark that, according to a most competent judge, Dr. Lyon Playfair, "the improvements suggested are happily conceived, and calculated to prove of immense benefit to the town; the spirit of the recommendations of the Commissioners of Inquiry, being completely carried through the whole Report." Although the proposed plan may be unexceptionable, yet all these partial efforts are, in principle, most objectionable; nothing but a general measure applicable to the whole kingdom, and superintended by responsible public officers, can reach the root of the mischief.

We have said that there is a willing Government; and among its members, happily, is that estimable nobleman, Lord Ebrington, whose zealous and personal labours in the advancement of this question are deserving of the highest praise. The whole of his lecture, in addition to a clear exposition of the causes of unhealthiness and on the means of removing it, abounds in the most elevated and benevolent sentiments. How would the whole constitution of society be renovated and ennobled if those who influence its progress, regulated their actions by the divine precepts of Christian charity, thus set forth.

"The golden rule of doing to others as we would be done by would never have led us into such wastefulness and extravagance as what you have seen. If we in the town and country, landlords and tenants, employers and employed, had endeavoured to make the material, moral, and spiritual condition of our neighbours as healthy as we would wish our own to be, we should have found our reward literally here upon earth. I have shown you the costliness of neglect; but in this as in all other cases we shall be deceived and led astray if we begin in a wrong spirit. If we seek merely that which is expedient, no foresight and calculation will be sufficient to guard us against error. Shrewd calculators enough there have been at Liverpool; but all their shrewdness and calculation has not prevented the waste of hundreds of thousands on ill health. Had one half of that energy and thought been devoted to their duty to their neighbour by that wealthy community, how much richer would they have been! 'Seek ye first the kingdom of God and his righteousness, and all these things shall be added unto you.'"—*Lord Ebrington's Lecture,* p. 37.

In alluding to that important point, the duty of the Government in this matter, it is encouraging to find Lord Ebrington thus expressing himself:—

"It only remains that I should say a few words of the power which Government has in this matter. Legislation can do much—very much—so much that no efforts of individuals or associations can avail without its help. We are dependent upon legislation for our supplies of water, and the construction of sewers. Unsound legislation may place a thousand obstacles in the way of both, but a good and comprehensive measure may carry these cheap blessings into every court and alley in the kingdom. To legislation, again, we must look for a good system of supervision and inspection, the abatement of nuisances, the closing up of crowded churchyards, the removal of cattle markets and slaughter-houses from the centre of our large towns, the consumption of smoke, the purification of our rivers, and the application of the valuable refuse of towns to its proper use, and what is doubtless more difficult, the regulation of the hours of work, and the enforcement of ventilation in public buildings, churches, schools, barracks, factories, shops, and workshops."—*L. c.*, p. 46.

It is still more fortunate that these sentiments are shared by other and influential members of the Administration, several of whom have evinced the warmest interest in the sanitary question.

The Lecture of Dr. Guy has reference to a very important subject—the feasibility of reducing the expense of improvement by the scientific application of the refuse of great towns to agricultural purposes; its great object being "to prove that the waste of life and health at present taking place is closely connected with a waste of the raw material of food." It is a truth important to be generally known "that unnecessary sickness and premature death impeach the prudence, no less than the humanity of the nation which suffers them, and that sanitary measures are, in every sense, and in every way, a gain."—*Lecture*, p. 8.

This proposition is established by various interesting details, but of which our space will only allow us to extract the following.

"Wherever a proper system of house-drainage prevails, the valuable excreta of the human frame containing the ashes of all the food that has been consumed by the inhabitants, find their way into the sewers. Experience has proved that these excreta, but especially the urine, are among the most effective of our manures; and that they far exceed in value the products of the farm-yard and all solid manures, not even excepting guano. It is well known too, that in China, and in those parts of the Continent of Europe where agriculture is most skilfully practised, great store is set by this fertilizing liquid.

"To this, the most important constituent of sewer-water, we must add, as also derived from house-drainage, the alkalies, potash and soda, which are so largely used for household purposes, in the form of pearl-ash, soap, and common salt. These alkalies form, as is well known, very important elements of the food and structure of plants.

"Such then, are the valuable matters poured into our sewers, wherever a proper system of house-drainage is in force.

"Large contributions are also made to the same fertilizing liquid by the refuse of slaughter-houses, markets, and manufactories. The animals fed and worked in our large towns also enrich the sewer-water, by that portion of their excreta which finds its way, in a more or less circuitous manner, into the sewers. Then we must not forget that our granite roads, rubbed down by constant traffic, furnish a large and valuable supply of silica, alumina, and iron, in a state of minute division, and therefore, ready to become the food of plants.

"I have yet to mention the large quantities of soot rich in ammonia and sulphurous acid, which, issuing from our chimneys, is brought down by every shower, and conveyed direct into the sewers, forming a not unimportant addition to their contents.

"I should not have entered so much into detail with regard to the contents of our sewers, but that I thought it of great importance to prove by every possible means the value of the fertilizing liquid which we are now so ignorantly wasting."—*Lecture*, p. 20.

So valuable are these debris of towns that it has been calculated the inhabitants of Chorlton-on-Medlock (a township of Manchester), rather less than 100,000 in number, would furnish sufficient nitrogen, phosphoric acid, and the other substances, to manure no less than 93,440 acres of wheat: and in Flanders, where these things are managed better than with us, the excreta of an adult are valued at £1. 17s. per annum. In an instance extracted from the Journal of Royal Agricultural Society, it is stated that some water-meadows at Clipstone Park, the property of the Duke of Portland, and which formerly were nothing but a swampy waste, producing for 300 acres but £80. a year, have yielded, by the application of sewage manure, as much as £11. 4s. per acre annually.

In conclusion, we would urge upon all whose sympathy has been touched by the unexampled sufferings of our poorer fellow-countrymen, as disclosed in the various reports and other documents which have from time to time appeared, to arouse themselves and give a practical direction to their philanthropy. The time is come for sustained and energetic action; public meetings, petitions to the legislature, appeals to members of parliament, these and similar measures are imperatively demanded of each and all who can exert any, the least, influence upon the march of public events. Whilst it is thus incumbent upon every class to make some effort, there are two professions upon whom, at this critical period, a heavy responsibility rests; we allude to the clergy and to medical practitioners. Daily witnessing, in all their appalling realities, the calamities and miseries inflicted upon the poor in our great towns and populous places; and knowing by personal experience the evils, which, owing either to a benighted ignorance or to a cruel selfishness, some individuals are unwilling to admit, the medical practitioner and the minister of religion have no excuse for inaction. That both will cheerfully enter upon their mission, the experience of the past will not permit us to doubt; and we may therefore confidently anticipate that the clamours which a few interested persons have already raised, will be instantly silenced by the united voice of science and religion, seconded by the good sense of the enlightened part of the community.

